# Association between sports type and overuse injuries of extremities in adults: a systematic review

**DOI:** 10.1186/s12998-017-0135-1

**Published:** 2017-01-13

**Authors:** Charlène Chéron, Christine Le Scanff, Charlotte Leboeuf-Yde

**Affiliations:** 1 CIAMS, Université Paris-Sud, Université Paris-Saclay, F-91405 Orsay, Cedex France; 2 CIAMS, Université d’Orléans, F-45067 Orléans, France; 3 Institut Franco-Européen de Chiropraxie, 72 Chemin de la Flambère, F-31300 Toulouse, France

**Keywords:** Cumulative trauma disorders, Overuse injuries, Sports type, Extremities, Epidemiology, Adults

## Abstract

**Background:**

Sports injuries are often described as overuse or traumatic. Little is known about the frequency of overuse injuries and, in particular, if they vary between different types of sporting activities.

**Purpose:**

To identify any differences between sports in relation to diagnoses of overuse injuries of the extremities (OIE) and anatomical areas most likely to be injured in adults and to compare these findings with those reported in youngsters, as identified in a previous review.

**Methods:**

A search was made in May 2015 and again in April 2016 in PubMed, SportDiscus, PsycInfo, and Web of Sciences. Search terms were « overuse injuries OR cumulative trauma disorders OR musculoskeletal injuries » AND « extremity OR limb » AND « physical activity OR sport OR risk factor OR exercises ». Inclusion criteria were: 1) prospective, or cross-sectional study design; 2) at least 1/3 of the population should be ≥ 19 years; 3) articles must clearly state if reported cases were classified as traumatic or overuse injuries in relation to a particular sports type, 4) sample size >50, and 5) articles must not deal with specific occupational subpopulations nor with clinical populations. A blinded systematic review was conducted and results reported per anatomical site of injury and diagnosis for the different sports.

**Results:**

In all, 10 of 1435 identified articles were included, studying soccer, beach-volleyball and triathlon. In general, the incidence estimates were low, never above 2.0/1000 h of practice, similar to results seen in children/adolescents. The incidence estimates and the diagnoses of OIE were given only in 4 articles on soccer, making comparisons between sports impossible. As in children/adolescents, the lower limb is more often affected than the upper but contrary to young people the injured site in adults is more often the knee and above, and there were also differences in the diagnoses for the two age groups.

**Conclusion:**

The literature does not permit to identify clearly the difference in the incidence of OIE for different sports showing that more but well-designed surveillance studies are needed.

## Background

Physical activity promotes the general well-being and has many direct health benefits [1–3]. Nevertheless, physical activity can also cause injuries that in turn may be responsible for reduced physical activities and even an inability to work. Moreover, these injuries may require medical care including surgery and perhaps long periods of rehabilitation. This may result in costs both on an individual and societal level.

Classically, injuries can be defined as traumatic or overuse depending on their etiology. An important prospective study following 1270 schoolchildren weekly by text-messages (and clinical examination if needed) regarding musculoskeletal injuries and physical activity brought a lot of information on musculoskeletal injuries. In order to study the epidemiology of musculoskeletal injury this method appeared to be more relevant than what is commonly seen in the literature in which data collection is usually performed in sports’ clubs, during a sporting event or using medical files. In this study it was found that overuse was a more common cause of reported injuries to the extremities than obvious trauma [4]. In addition, it was noted that the lower extremities were more commonly injured than the upper extremities.

A recent literature review on the link between overuse injuries of extremities (OIE) and specific types of sport in children and adolescents concluded that it was not possible to determine and compare the incidence of OIE between sports due to methodological heterogeneity of studies [5]. Although, in general, the most commonly injured sites are the knee and the heel [4], the risk of reported injury differed somewhat between sports in relation to anatomical site. Interestingly, sports that put a lot of strain on the upper extremity, such as handball and volleyball resulted in overuse injuries of the lower extremity at least as often as of the upper extremity. It was also noted that the three most common diagnoses of OIE are tendinitis/bursitis, strain and osteochondral disorders across all sports [4] and these do not change between sports [5]. Unfortunately, articles often did not report clearly exact site and diagnosis of injuries.

The skeleton of children and adults do not have the same consistency and maturity, so this information relating to children may not be applicable to adults. To our knowledge, no clear information is available on sports-related OIE for the adult population.

For this reason, we conducted a systematic review to gain a better understanding of sports-specific OIE in adults with three objectives:

1. To determine the incidence of OIE for various sports
2. To identify any differences between sports in relation to the anatomical areas most likely to be injured
3. To identify any differences between sports in relation to diagnosis

To be able to compare the findings on adults to those in children, we used a similar method to our previous review on children and adolescents [5].

## Methods

### Systematic literature search

A first search was performed in May 2015 and a final search in April 2016 in PubMed, SportDiscus, PsycInfo, and Web of Sciences using the search terms « overuse injuries OR cumulative trauma disorders OR musculoskeletal injuries » AND « extremity OR limb » AND « physical activity OR sport OR risk factor OR exercises » in different combinations (MeSH terms and free text). An additional citation search of reference lists of the retrieved articles was performed. No restrictions were placed on date of publication and no attempts were made to search the grey literature.

### Inclusion criteria

We used the Preferred Reporting Items for Systematics reviews and Meta-Analysis (PRISMA) guidelines in this review [6]. The first author applied the inclusion criteria to the title and abstract of the articles identified as possible relevant research articles from the literature search. Full-text screening was then done by two authors independently of each other to determine which articles should be included in the review. Inclusion criteria were: 1) a study design that was prospective or cross-sectional; 2) at least 2/3 of the study population should consist of ≥19 years olds or results should be reported specifically for different age groups. To determine this we looked for information on the range age, the mean age with the standard deviation, and the proportion of adults, when data were reported for age groups. In study samples consisting of “professionals” but no further information of age, we assumed that these would consist mainly of adults; 3) the article must state clearly if reported cases were classified as traumatic or overuse injuries in relation to a particular sports type; 4) a sample size greater than 50; and 5) the article must not deal with specific occupational subpopulations (such as military) nor with clinical populations. Only articles in English, French or a Scandinavian language were considered, as the authors could read these languages.

### Data extraction

The checklists were extracted from a previous review on OIE and sports’ type on children and adolescents [5]. We used two descriptive checklists, one quality checklist and three tables of results [5].

Table 1 included information on the first author, year of publication, type of sport and level (recreational or elite). Moreover, we reported the number of subjects invited, the number and age of participants, the duration and the method of data-collection, and a description of the person who collected the information and/or diagnosed the injury.

**Table 1:** Characteristics of study participants in 10 reviewed studies on overuse injuries of the extremities in adults

Sport	First author Year Country of study	Sport participation level	N participants/ N invited	Sex	Age (Min-Max) Mean ± SD	Duration of data collection & follow-up frequency (N/time)	Method of data collection	Data source
Soccer	Kristenson 2013 [10] Sweden	Elite	1507/?	M	(?) Group 1: 25.2 ± 5 Group 2: 25.0 ± 5	2 seasons (?)	Standardized forms	Medical person for each team
	Tegnander 2008 [11] Norway	Elite	181/?	F	(17–34) 23 ± 4	1 season (?)	Not described in the text but based on 2 references^a^	The team physiotherapists
	Jacobson 2007 [12] Sweden	Elite	269/?	F	(16–36) 23 ± 4	1 season (Weekly)	-Standardized attendance protocol -Interviewed telephone: standardized protocol	Physiotherapists of the team and medical personal telephone interview of athlete
	Lüthje 1996 [13] Finland	Elite	263/263	M	(17–35) age group reported ?	1 season (?)	Physical exam	Team physician
	Nielsen 1989 [14] Denmark	Recreational	123/?	M	(>16) age group reported ?	1 season (Weekly)	Examination recorded on special card	Medical doctors
	Eirale 2013 [15] Qatar	Elite	230/?	M	(?) 28,4 ± 4.4	1 season (Daily)	Standard injury cards	Medical staff of club
	Faude 2005 [16] Germany	Elite	149/?	F	(?) 22.4 ± 5.0	1 season (Weekly)	Diary	Medical staff & coach
	Ekstrand 2011 [17] Sweden	Elite	767/?	B	(B: 15–38) M: 25 ± 5 F: 23 ± 4	5 seasons (Monthly updates)	Standard injury form	Medical staff
Triathlon	Andersen 2013 [19] Norway	Elite	174/274	B	(?) 38 ± 9	26 weeks (Every 2nd week)	Questionnaire	Athletes
Beach Volleyball	Bahr 2003 [18] Australia, Norway, Portugal	Elite	?/?	B	(?) ? Professional players	5 championship (?)	Standardized forms	Medical staff

? : Information not provided

*F* female

*M* male

*B* both

^a^Orchard J, Orchard Sports injury classification system (OSICS). Sport Health 1993; 11:39–41. Fuller CW, Ekstrand J, Junge A, Andersen TE, Bahr R, Dvorak J, et al. Consensus statement on injury definitions and data collection procedures in studies of football (soccer) injuries. Br J Sports Med. 2006;40:193–201

Table 2 specified the criteria used in the article to define “injury” and “overuse injury” inspired respectively by Bahr [7] and Fuller [8]. The criteria for “injury” were: sport-related, complaint, time-loss, and medical attention. Regarding the definition of “overuse” we used: 1) repeated micro trauma, 2) no single, identifiable cause; 3) activity exceeds tissue tolerance and 4) gradual onset. Because some articles used other criteria, we added the column “other”. For a discussion of the rationale behind these definitions, please see our previous publication, where this is explained in detail [5].

**Table 2:** Criteria used to define injury and overuse injuries in the 10 articles on overuse injuries of the extremities in adults

Sports	Authors Year	Criteria for injury				Criteria for classified injury as overuse				
		Sport-related	Complaint	Time-loss	Medical attention	Repeated micro trauma	No single, identifiable cause	Activity exceeds tissue tolerance	Gradual onset	Other
Soccer	Kristenson 2013 [10]	X		X			X		X	
	Tegnander 2008 [11]	X		X			X		X	
	Jacobson 2007 [12]	X		X			X			
	Lüthje 1996 [13]	X		(X)	X		X			“Pain syndrome of musculoskeletal system appearing during physical exercise without any known trauma, disease, deformity or abnormality that might have given previous symptom. The symptoms started during physical exercise and were located as a previously symptom-free region of the body. Temporary muscle pains associated with increasing training were not recorded in the study. The diagnosis of an overuse injury was made on the basis of medical history and a thorough medical examination. “
	Nielsen 1989 [14]	X		X						“Strains were considered to be acute overuse injuries. “ “Some of the overuse injuries (tendinitis/synovitis) occurred under unknown circumstances.”
	Eirale 2013 [15]	X		X		X	X			
	Faude 2005 [16]	X		X		X				
	Ekstrand 2011 [17]	X		X			X		X	
Beach volleyball	Bahr 2003 [18]			X or	X or				X	
Triathon	Andersen 2013 [19]	X								“Overuse injury” is not defined anywhere but the article was included in the review because it uses this term in the title.
Total		9/10	0/10	9/10	2/10	2/10	6/10	0/10	4/10	

The quality checklist can be seen in Table 3. It was reported in this table if 1) the participation rate was stated (or could be calculated), 2) the injury was diagnosed by a health professional, 3) the diagnosis and anatomical site were clearly and completely reported, 4) the incidence of OIE was reported, and 5) if the number of injuries could be reported in relation to number of hours of exposure and individuals.

**Table 3:**
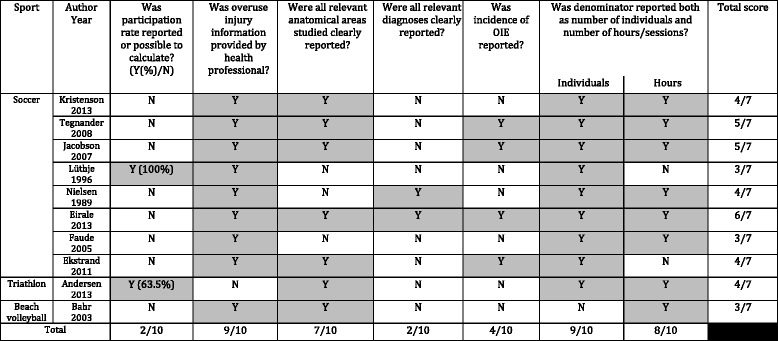
Quality checklist of methodological aspects of 10 studies on overuse injuries of the extremities (OIE) in adults

*N* no

*Y* yes; when positive answers have been highlighted

Three evidence tables reported the findings. Table 4 reported the estimates of rates of OIE. The incidence was included if it was clearly reported in the article. Moreover, we calculated the proportion of OIE based on the total number of hours of exposure and reported this as number of injuries per 1000 h of exposure.

**Table 4:** Incidence and proportion of overuse injuries of the extremities (OIE) based on numbers of hours of exposure in 10 studies on adults

Sport	Author Year	Number of OIE	Incidence estimate given in the article	Number of hours of exposure	Proportions of OIE based on number of hours of exposure (*1000)
Soccer	Kristenson 2013 [10]	406	-	367490	1.10
	Tegnander 2008 [11]	21	0.8 per 1000 game hours 0.7 per 1000 training hours	30619	0.68
	Jacobson 2007 [12]	62	Between 0.0 to 0.6 depending on area	47075	1.32
	Lüthje 1996 [13]	16	-	-	-
	Nielsen 1989 [14]	30	-	15908	1.89
		79	-	23400	3.38
	Eirale 2013 [15]	115	From 0.03 to 2.0 (varying depending on diagnosis & localisation)	39100	2.94
	Faude 2005 [16]	7	-	39162	0.18
	Ekstrand 2011 [17]	?	From 0 to 0.5 (depending on diagnosed area)	M: 198071 F: 48404	-
Triathlon	Andersen 2013 [19]	403	-	48024	8.39
Beach volleyball	Bahr 2003 [18]	21 Estimates from diagram	-	1576	13.32

* multiplied by 1000

In Table 5 the numbers of OIE were listed by anatomical area. We highlighted those two that were most commonly reported. Table 6 showed the same type of information but based on the diagnosis.

**Table 5:**
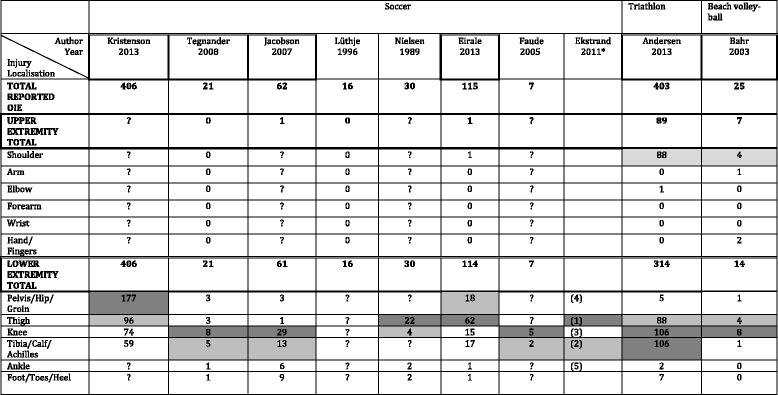
Site of overuse injury of the extremities by sports in 10 studies on adults

The two most common injury sites in each article are highlighted: Like this for the most common and like this for the second most common

“?”= Information not provided

OIE: Overuse injuries of extremities

Articles in which all OIE are described and in which all the sites of OIE are clearly described are framed, i.e. Author/Year

*: the number of injuries was not reported in this article but we have the incidence so we could rank the localisation. 1 means the most often reported, 2, the 2nd most often, and so on

**Table 6:**
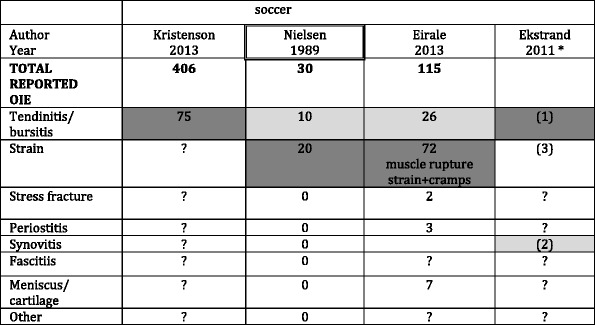
Injury diagnosis according to sports type for 4 studies on adults that included specific diagnosis

OIE: Overuse injuries of extremity

The two most common diagnoses in each article are highlighted: Like this for the most common and like this for the second most common

Six articles have been excluded in this table because they did not mention any diagnosis:

Articles in which all OIE are described and in which all the diagnosis of OIE are clearly described are framed, i.e. Author/Year

*: The number of injuries was not reported in this article but we have the incidence so we could rank the diagnoses. "1" means the most often reported, "2" the 2nd most often, and so on

The AMSTAR checklist [9] was used as a guide for this review. However, tests for homogeneity and publication bias were not carried out because no such statistical information could be extracted to be used in this review. Furthermore, articles were not screened for conflict of interest statements, as this aspect was irrelevant for the current topic (no obvious financial gains).

### Review process and interpretation of data

Two of the authors extracted the information separately and blind to each other’s findings. Their findings were compared to detect extraction errors. The third author was available for arbitration in case of disagreements between the two reviewers. The quality data were used for descriptive purpose only and to provide a basis for research recommendations.

The review was registered in the PROSPERO database: CRD42015032477.

## Results

### Number of articles

**Fig. 1:**
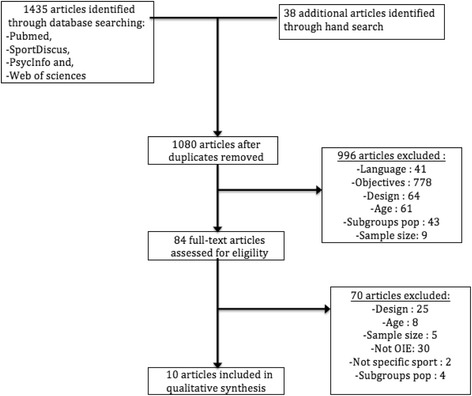
PRISMA flowchart showing selection of articles

Initially, on the basis of the database and citation searches, 1435 articles were identified, leaving 1080 articles after duplicates were removed. Of these, only 10 were retained after scrutiny of their title, abstract and full-text. The criteria of non-inclusion of the articles are presented in Fig. 1. Most of the excluded studies did not deal with specific sports or OIE. Although it often was difficult to extract some of the data, it was never necessary to use the arbitration process.

### Study design, participants and method

Three sports were covered in the 10 articles included in this review: soccer [10–17], beach volleyball [18], and triathlon [19]. They were published from 1989 to 2013 and nine were conducted in Europe. The design was prospective for all studies except for one [18], which combined a prospective and retrospective study, but only results from the prospective study were used in this review. In all studies, the study samples were obtained from sports clubs or at competitions. The level of sport participation of the study participants varied from recreational to elite level, but for the majority of articles it was at an elite level.

The number of participants ranged from 123 to 1507 (Table 1). Four studies included only men, three studies only women, and three studies both sexes (Table 1). The age of participants was not clearly described in all articles, but when it was, it varied from 15 to 39 (Table 1), with the mean age of 23 to 38 years.

The duration of data collection, when described, ranged from 26 weeks to 2 seasons and for one article it took place during five championships. The frequency of follow-ups within this duration, when reported, was often weekly (Table 1).

### Definition of overuse sport injuries

The definition of ‘sports injury’ differed between articles (Table 2). Most commonly, a case was defined by time-loss and was nearly always depending on a link to the specific sport activity studied. The specific definition of overuse injury was most commonly based on the concept of the absence of a single, identifiable (traumatic) cause (*N* = 6), followed by gradual onset (*N* = 3), repeated micro-trauma (*N* = 2), and combinations thereof. Nobody stated explicitly that the activity had to exceed tissue tolerance, although this probably would have been taken into consideration during medical examination.

### Quality of the studies

Although method sections in this type of studies often are very similar, specific information was sometimes difficult to obtain for our purposes. As can be seen in Table 3, response rates were often unreported, as well as incidence estimates. Therefore, it became necessary to calculate the proportion of cases based on exposure, which explains the last column in the quality checklist. In relation to outcomes, overuse injuries per anatomical area and diagnosis were often not systematically reported. On the positive side, health professionals were usually responsible for the data collection.

### Incidence estimates of overuse injuries

The incidence estimates of OIE are shown in Table 4. These were reported in only four articles dealing with soccer but they all reported it differently.

Tegnander et al. [11] calculated the incidence distinguishing training from game exposure. Moreover, they reported the incidence for OIE in general with incidence estimates of OIE being 0.8 per 1000 h of game and 0.7 per 1000 h of training.

The other three articles reported the incidence based on 1000 h of sport participation. Jacobson et al. [12] provided the incidence for OIE based on the area injured, which varied from 0 for the hip, groin and thigh to 0.6 for the knee. Eirale et al. [15] provided the incidence for various diagnoses and localisations. Regarding the diagnoses, the incidence varied from 0.1 for fracture and synovitis/periostitis to 2.0 for muscle rupture/cramps. Regarding the localisation, the incidence estimates varied from 0.03 for shoulder, ankle and foot/toe to 1.7 for the thigh. Ekstrand et al. [16] reported the incidence for the most common OIE subtypes while combining the diagnosis and the localisation. The incidence varied from 0.03 for the ankle joint synovitis and calf muscle cramp/spasm to 0.5 for hamstring overuse/hypertension.

### Proportion estimates of overuse injuries

Table 4 shows also the proportion of OIE based on exposure. It could be calculated in 8 articles and varied from 0.18 to 13.32 per 1000 h of exposure. The two studies that did not study soccer reported higher proportion of OIE than the others. Methodological differences could probably explain these results.

### Injury site and diagnosis in general

The lower limb was most often affected (Table 5) and especially the knee, tibia, thigh and pelvis/hip/groin.

Only few articles described the diagnosis of overuse injury. For that reason, only 4 articles could be included in Table 6. The most frequently provided diagnoses were tendinitis/bursitis, and strain.

### Differences in overuse injuries according to sports type

For all sports covered, the lower limb was more often affected than the upper limb. Again, it was impossible to compare the incidence rates between sports, because it was only reported in the articles on soccer. When considering the proportion of OIE per 1000 h of exposure, different results are found. For soccer, this proportion is <3.5 (and often around 1), 8 for triathlon, and 13 for beach volleyball. However, methodological considerations could well explain these differences.

In soccer, the pelvis/hip/groin appeared to be more commonly affected than in the two other sports.

We could not compare the diagnosis of OIE between sports because only articles on soccer reported the diagnosis.

## Discussion

### Summary of findings

This appears to be the first systematic review on OIE in adults comparing the occurrence in various sports. We attempted to identify any differences between sports in relation to diagnoses and anatomical areas most likely to be injured. We were able to retrieve 10 studies on three different sports: soccer (*N* = 8), triathlon (*N* = 1) and beach volleyball (*N* = 1). Methodological differences between studies and a limited number of studies and sports studied made it difficult to provide clear answers. However, in relation to the proportion of OIE it varied between 0.2 to 13.3 per 1000 h of exposure, with soccer not having the highest estimates. This proportion is generally more important in adults than in youngsters, where results around 0.5 were found [5].

Injury site was, as for the youngsters [5], mainly the lower limb. However, in adults this was reported rather for the knee, tibia, thigh and pelvis/hip/groin whereas in youngsters it was the knee and the lower leg.

As for the diagnoses, they were most frequently (when at all provided) reported to be tendinitis/bursitis, and strain, whereas in children and adolescents the most commonly reported diagnoses were tendinitis/bursitis and periostitis [5].

### Methodological aspects of the articles reviewed

A large body of literature on sports injuries of adults, as well as of children, is written by a group of researchers that uses the same methodological approach when surveying injuries in different sports. Typically, they study injuries in single sport clubs or during specific sports events with the ultimate goal to compare risk estimates for various sport activities. To record a sufficiently large number of injuries of specific sports in the general population is of course difficult, hence this approach. However, when choosing such a tactic, it would be relevant to collect similar data from several clubs/events, in order to even out any bias associated with single convenience samples of such type.

After having reviewed this literature on both children/adolescents and adults, it is clear that even when multiple studies are found for similar sports, data are often collected at different intervals, in different ways, using different definitions for injury, and for different specific types of injuries. Authors do not clearly report diagnosis and anatomical areas of injury, and if they do, they often leave out the one or the other. This, also, makes it difficult to make comparisons and to establish risk estimates. A simple example is the difference in estimates expected when the presence of an “injury” is reported as “complaint”, as “sought care”, or “time loss”. Further, in the case of “overuse”, absence of a traumatic etiology seems often automatically to result in a diagnosis of an “overuse” injury, merely because the person with the complaint was involved in a sporting activity. It is not logical that people involved in studies on sport injuries only have these two possible diagnoses, traumatic or overuse injury. Surely patients from the general population are diagnosed from a larger spectrum of possibilities. Clear criteria for this diagnostic label have been proposed [5] and discussed in the literature [20], but seem to be largely ignored, at least when reports are written up.

As for the definitions of “incidence” and “prevalence”, true incidence and prevalence estimates are usually not distinguished in studies within this area. The incidence is defined as number of injuries based on 1000 h of session (training, competition or both), in general without regards concerning the previous injury. In fact, this should not really be called incidence but prevalence. This issue has been previously discussed by Bahr [7]. Further, the numbers of potential and included study subjects are often not reported. Clearly, an injury rate (per 1000 h) would be more credible when obtained from many study subjects than from a few. It would therefore be useful for the reader to have access to both these denominators.

Admittedly, the objectives of our review were not the same as the objectives of the studies under review, which makes difficult the extraction of information in our review. Nevertheless, as we have already discussed in our previous review on children/adolescents [5] in our opinion, this research area would benefit from a well-reasoned consensus approach to the various definitions.

### Methodological aspects of our review

Our review followed the current guidelines, using a transparent approach, searched several databases, and data were extracted blindly by two reviewers. However, it is possible that some articles could have been missed, as only texts written in English, French and Scandinavian languages were acceptable for inclusion. Checklists for data extraction have been previously tested and used in a previous review and were therefore known to be user-friendly and relevant.

Sometimes we had to make assumptions regarding the nature of injuries, when exact information regarding the site of injury was missing. Thus two diagnoses, tendinopathy and periostitis, were systematically considered as extremity injuries, whereas some diagnoses such as strain was not, because it could affect the spine.

### Discussion of findings regarding the incidence of OIE

We did not find any information in the literature on OIE in the general population of adults. However, the incidence of OIE in general population of schoolchildren has been reported to be 2.3(1.6–3.0 95% CI) for the upper extremity and 3.7(3.5–4.0) for the lower extremity [4].

### Discussion of findings regarding the anatomical site of OIE

As observed in the previous review on children and adolescents [5], the lower extremity is more often affected than the upper extremity in the sports studied. Only three sports could be considered in this review, so it is difficult to compare the localisation of OIE between sports. However, we noted that in soccer, in youngsters and in adults, the pelvis/hip/groin are more often affected than in the other sports. We assumed that this is due to the shearing force often imposed on the pelvis in soccer.

### Discussion of findings regarding the diagnosis of OIE

Only four articles provided good information on the diagnosis of OIE and they all studied soccer, making it impossible to compare this finding with other sports. In childhood, 8 articles reported the diagnosis making a comparison relevant. However, for all sports covered, it was always the two same diagnoses that were reported.

Tendinis/bursitis is the most common diagnosis both in childhood and adulthood, followed in adults by synovitis, and in youngsters by periostitis. Probably because of the difference in bone skeletal maturity, osteochondral disorders, present in youngsters, did not appear in adults.

## Conclusion

This research area suffered from lack of information because of few relevant studies and methodological problems, which makes difficult the extraction and comparison of the incidence of OIE in relation to both their diagnosis and localisation. However, we could conclude that the incidence of OIE is low in adulthood, as it was previously found to be in childhood, across most studies reviewed. The localisation of OIE seems to be predominantly in the lower limb, with some differences relating to exact anatomical area between sports. Obviously, the search for risk sports and specific types of injuries needs to be undertaken in a more systematic and homogeneous manner, to make the information useful for the purposes of prevention.

## Abbreviation

OIE: Overuse injuries of extremities
